# Capsaicin directly promotes adipocyte browning in the chemical compound-induced brown adipocytes converted from human dermal fibroblasts

**DOI:** 10.1038/s41598-022-10644-8

**Published:** 2022-04-22

**Authors:** Yukimasa Takeda, Ping Dai

**Affiliations:** Department of Cellular Regenerative Medicine, Graduate School of Medical Science, Kyoto Prefectural University of Medicine, 465 Kajii-cho, Kawaramachi-Hirokoji, Kamigyo-ku, Kyoto, 602-8566 Japan

**Keywords:** Cell biology, Molecular biology

## Abstract

Human brown fat is a potential therapeutic target for preventing obesity and related metabolic diseases by dissipating energy as heat through uncoupling protein 1 (UCP1). We have previously reported a method to obtain chemical compound-induced brown adipocytes (ciBAs) converted from human dermal fibroblasts under serum-free conditions. However, pharmacological responses to bioactive molecules have been poorly characterised in ciBAs. This study showed that the treatment with Capsaicin, an agonist of transient receptor potential vanilloid 1, directly activated adipocyte browning such as UCP1 expression, mitochondrial biogenesis, energy consumption rates, and glycerol recycling in ciBAs. Furthermore, genome-wide transcriptome analysis indicated that Capsaicin activated a broad range of metabolic genes including glycerol kinase and glycerol 3-phosphate dehydrogenase 1, which could be associated with the activation of glycerol recycling and triglyceride synthesis. Capsaicin also activated *UCP1* expression in immortalised human brown adipocytes but inhibited its expression in mesenchymal stem cell-derived adipocytes. Altogether, ciBAs successfully reflected the direct effects of Capsaicin on adipocyte browning. These findings suggested that ciBAs could serve as a promising cell model for screening of small molecules and dietary bioactive compounds targeting human brown adipocytes.

## Introduction

Obesity is caused by a chronic energy imbalance between energy intake and expenditure^1^. It is a major risk factor for cardiovascular and metabolic diseases, including type 2 diabetes. Owing to the obesity pandemic, new therapeutic interventions that can safely enhance energy expenditure are in demand to combat it. White adipocytes are specialised for the storage of excess energy in the form of triglycerides, whereas brown adipocytes produce heat by dissipating energy to resist hypothermia^2–4^. Uncoupling protein 1 (UCP1), a brown adipocyte-specific protein located in the mitochondrial inner membrane, uncouples fatty acid oxidation from mitochondrial production of adenosine triphosphate (ATP)^5^. The activation of human brown adipose tissue (BAT) by cold exposure reportedly improves whole body energy expenditure and systemic glucose homeostasis^6–8^. The prevalence and activity in human BAT are associated with a lower body fat mass, decreased age, and higher plasma docosahexaenoic acid levels^9^. The clinical significance has been well studied in murine brown/beige adipocytes^10^. Expanded beige adipocyte population in white adipose tissue (WAT) was associated with increased energy expenditure, body weight loss, and improved insulin sensitivity. Recently, a retrospective study using ^18^F-fluorodeoxyglucose positron emission tomography–computed tomography scans in a large cohort suggested that brown fat improves the levels of blood glucose, triglycerides, and high-density lipoproteins^11^. The study has also demonstrated that brown fat is associated with a lower risk of type 2 diabetes, dyslipidemia, coronary artery disease, cerebrovascular disease, congestive heart failure, and hypertension, indicating the potential of brown fat to improve cardiometabolic health in humans^11^. Therefore, we expect that identifying drugs and dietary compounds that enhance brown fat activity and adipogenesis may foster a safe and effective nutritional intervention strategy to prevent obesity.

Several food ingredients exert anti-obesity effects through the activation of Transient-receptor potential vanilloid 1 (TRPV1)^12^, a known receptor of Capsaicin, the principal pungent ingredient in chili peppers^13^. Accumulating evidence has suggested that prolonged administration of either Capsaicin or Capsinoids, non-pungent Capsaicin analogues, for several weeks resulted in beneficial effects on fat oxidation, energy expenditure, insulin sensitivity, and body fat mass in both rodents and adult humans^14–17^. Although the mechanism of action of Capsaicin involves multiple tissues, its anti-obesity effects are mediated by the activation of the sympathetic nervous system via TRPV1 on sensory neurons^18–22^. Norepinephrine (NE) secreted at the end of sympathetic nerves increases cellular levels of cAMP via β-adrenergic receptors, which overlaps with cold-induced thermogenesis pathway. cAMP activates CREB and ATF2, the downstream transcription factors, to activate a series of thermogenic genes including *Ucp1*^23^. Besides Capsaicin and Capsinoids, other dietary compounds such as eicosapentaenoic acid and 10-Oxo-12(Z)-octadecenoic acid similarly exerted anti-obesity effects via the activation of TRPV1^24,25^. In contrast, a limited number of studies have shown direct effects of Capsaicin in metabolic tissues such as liver, kidney, pancreas, gastrointestinal tract, WAT, and BAT, where TRPV1 is expressed^26,27^. For example, treatment with Capsaicin in murine 3T3-L1 adipocytes suppressed adipogenesis and decreased triglyceride accumulation^28,29^, although the high-dose regimen increased lipid accumulation^30^. In HB2 immortalised mouse brown preadipocytes, TRPV1 expression was induced during adipocyte differentiation^31^. Capsaicin addition in the early stage of the differentiation did not affect the transcription of adipogenic genes such as *Fabp4*, *Ppargc1a*, and *Pparg*, whereas the treatment in the late stage slightly activated their expression. In both experimental conditions, *Ucp1* expression was not changed and the induction of *Ucp1* by Forskolin was decreased. In a subsequent study, the same group showed that the treatment with Capsaicin at a supra-pharmacological concentration activated the expression of *Ucp1*, *Ppargc1a*, and *Elovl3* along with efficient differentiation and lipid accumulation in HB2 adipocytes^32^. However, the treatment with a strong TRPV1 antagonist, 5′-Iodoresiniferatoxin, did not inhibit Capsaicin-induced *Ucp1* expression and lipid accumulation, suggesting that these effects were TRPV1-independent. The authors of the study suggested that endoplasmic reticulum stress caused by the supra-pharmacological concentration was responsible for Capsaicin-induced brown adipogenesis in HB2 adipocytes. Thus, the positive effects of Capsaicin have remained unclear, especially in human brown adipocytes.

We have previously developed a technique for direct conversion from human dermal fibroblasts into chemical compound-induced brown adipocytes (ciBAs) under serum-containing or serum-free culture conditions^33–35^. Furthermore, in a recent study, we have shown that ciBAs undergo a broad range of transcriptional changes that resemble adipocyte browning in adipose tissue-derived mesenchymal stem cells (AdMSCs)^36^. One of the most characteristic differences between them is the expression pattern of major uncoupling proteins. ciBAs expressed UCP1 at a higher level than the AdMSC-derived adipocytes which predominantly expressed UCP2, indicating that ciBAs might serve as a promising model for human brown adipocytes. The availability of human primary brown fat is limited owing to its scarceness, the fragile nature, and an invasive procedure to obtain biopsies^37^. In this context, an appropriate in vitro model for human brown/beige adipocytes is required for basic research, drug screening, and future clinical use. Therefore, in this study, we aimed to analyse the direct effects of Capsaicin as an agonist of TRPV1 on adipocyte browning in ciBAs. Furthermore, we verified whether the response in ciBAs was valid by comparing the response in the AdMSC-derived adipocytes.

## Results

### Capsaicin enhances UCP1 expression and efficiency of the direct conversion into ciBAs

**Figure 1:**
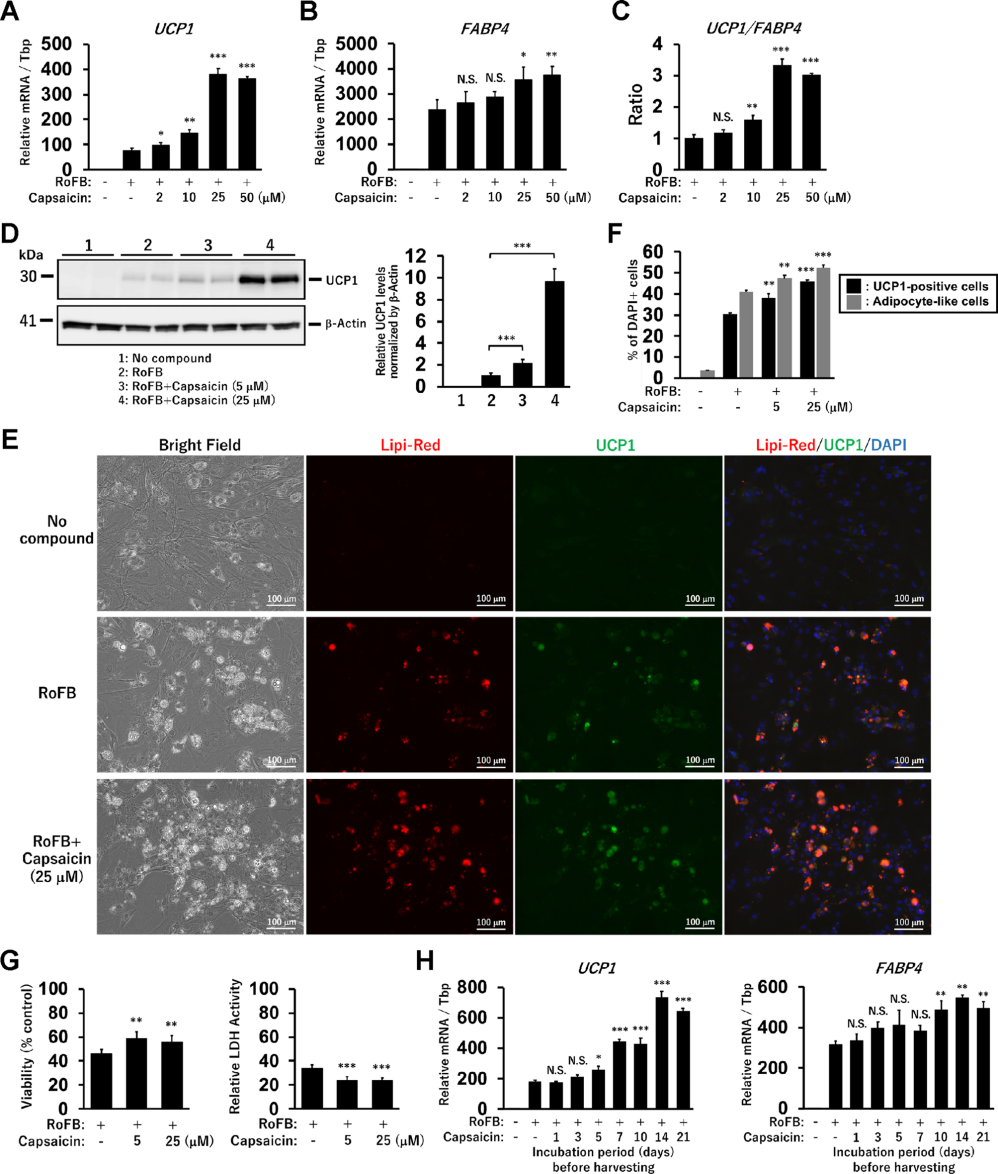
Capsaicin enhances UCP1 expression in ciBAs. (**A,B**) The expression of *UCP1* (**A**) and *FABP4* (**B**) was quantified by qRT-PCR analysis in ciBAs induced in the presence of Capsaicin at different concentrations as indicated. (**C**) Adipocyte browning activated by Capsaicin was evaluated by the ratio of *UCP1* to *FABP4* expression. (**D**) UCP1 protein was detected by immunoblotting analysis. The band intensities were quantified by densitometry using ImageJ software. β-Actin was used as a loading control for normalisation. (**E**) Representative images of bright field, Lipi-Red staining (red), UCP1 expression (green), and merged image in the control cells, ciBAs, and Capsaicin-treated ciBAs at 25 μM. The nuclei were visualised by DAPI (blue). Scale bars represent 100 μm. (**F**) To evaluate the conversion efficiency, the percent ratio of adipocyte-like cells with lipid droplets and UCP1-positive cells were calculated. (**G**) Cell viability was measured by the WST-8 regent in ciBAs and Capsaicin-treated ciBAs at 5 μM and 25 μM for 3 weeks. Cytotoxicity was assessed by measuring lactate dehydrogenase (LDH) activity in the culture supernatants. (**H**) The expression of *UCP1* and *FABP4* was measured in ciBAs treated with Capsaicin at 25 μM for different incubation periods (days) before harvesting the cells on Day 21 for subsequent qRT-PCR analysis. Data represent mean ± SD (n = 3). Student’s *t*-test: *P < 0.05, **P < 0.01, ***P < 0.001, N.S.; not significant.

ciBAs were converted from human dermal fibroblasts by a serum-free brown adipogenic medium (SFBAM) including the chemical cocktail (RoFB) consisting of Rosiglitazone, Forskolin, and BMP7 as previously reported^34^. To evaluate the pharmacological response through TRPV1 in ciBAs, Capsaicin was additionally treated during the direct conversion. The expression of the brown adipocyte-specific marker, *UCP1*, was increased in the presence of Capsaicin at concentrations higher than 2 μM, and reached to its highest at 25 μM obviously (Fig. 1A). The expression of an adipocyte-enriched gene, *FABP4*, was also moderately increased at concentrations of 25 and 50 μM (Fig. 1B). The ratio of the expression of *UCP1* to *FABP4* was used as an indicator to evaluate the effects of Capsaicin on adipocyte browning in ciBAs (Fig. 1C). The ratio was approximately three times higher at 25 μM than that of the Capsaicin-untreated control. Immunoblotting analysis showed that UCP1 protein levels were induced at a concentration of 25 μM (Fig. 1D). Furthermore, immunocytochemical analysis indicated that cells stained with Lipi-Red expressed UCP1 in both ciBAs and Capsaicin-treated ciBAs (Fig. 1E). The increased number of UCP1-positive and adipocyte-like cells indicated that Capsaicin also enhanced the conversion efficiency (Fig. 1F). Capsaicin treatment at 5 and 25 μM for 3 weeks enhanced cell viability and reduced cytotoxicity (Fig. 1G). Transient treatment with Capsaicin for a few days before harvesting had almost no effect on the expression of *UCP1* and *FABP4* (Fig. 1H). Their expression was increased by the incubation for more than 7 days, suggesting that prolonged treatment was required to elicit the effects of Capsaicin to activate *UCP1* in ciBAs.

### Cellular mitochondrial levels and respiration in Capsaicin-treated ciBAs

**Figure 2:**
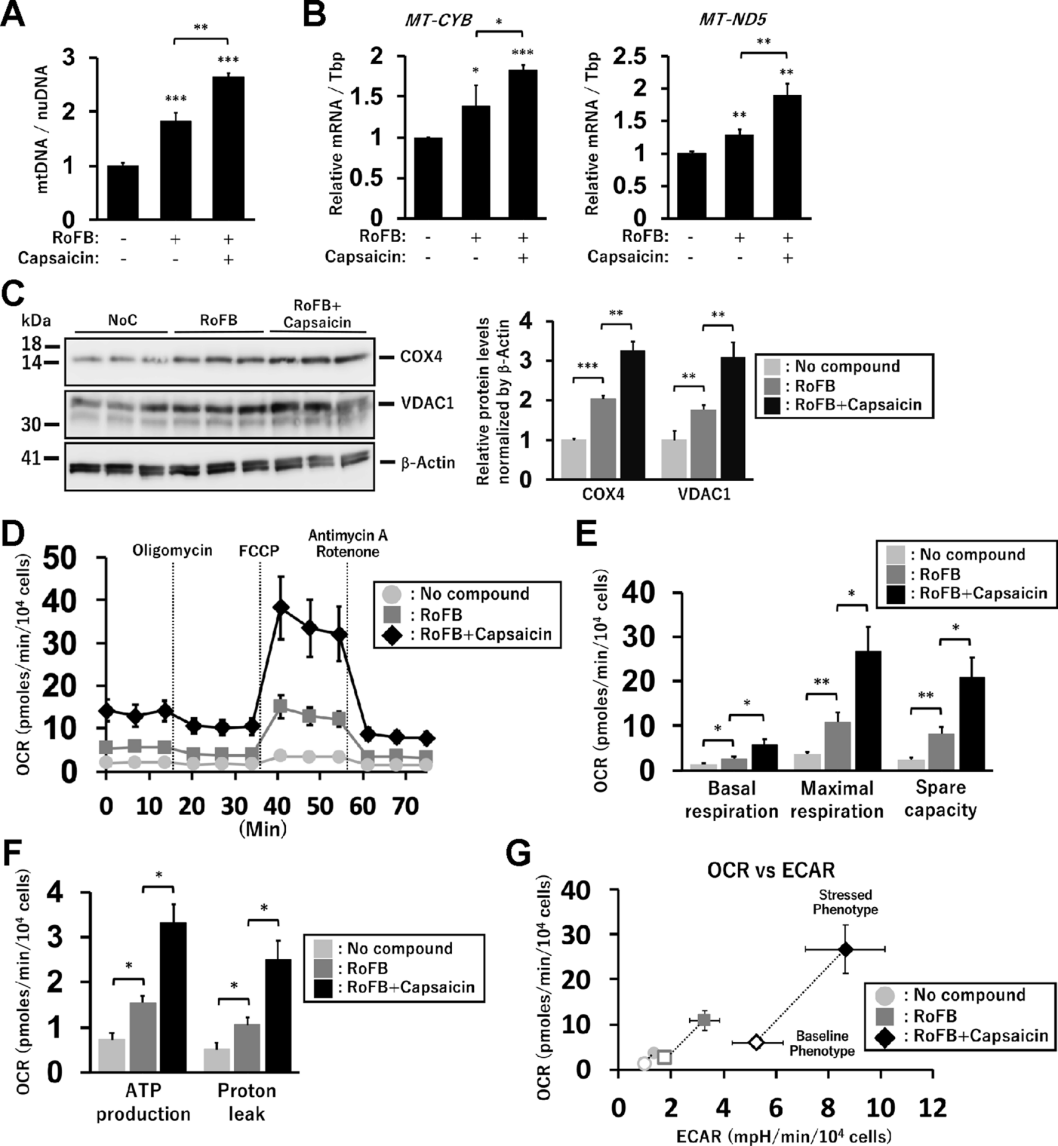
Measurement of mitochondrial levels and OCR in Capsaicin-treated ciBAs. (**A**) The relative ratio of mitochondrial DNA (mtDNA) to nuclear DNA (nuDNA) was determined by qPCR analysis in the control, ciBA, and Capsaicin-treated ciBAs. (**B**) The expression of mitochondria-encoded genes, *MT-CYB*, and *MT-ND5*. (**C**) Immunoblotting of mitochondrial marker proteins, COX4 and VDAC1. The band intensities were quantified by densitometry using ImageJ software and normalised by β-Actin loading control. Data represent mean ± SD (n = 3). (**D**) Oxygen consumption rate (OCR) was measured using Seahorse XFe96 extracellular flux analyzer in the control cells incubated with no compound (light grey circles) and ciBAs induced by either RoFB (grey squares) or RoFB + Capsaicin (black diamonds). Mitochondrial respiration inhibitors, oligomycin, FCCP, and antimycin A/rotenone, were added during the measurement as indicated. (**E,F**) OCR corresponding to basal respiration, maximal respiration, spare capacity (**E**), ATP production, and proton leak (**F**) was compared between either Capsaicin-treated or -untreated ciBAs. (**G**) Energy phenotype profile in the control cells (light grey circles) and ciBAs induced by either RoFB (grey squares) or RoFB + Capsaicin (black diamonds). OCR and extracellular acidification rate (ECAR) are plotted under basal (open circle, square, and diamond) and stressed (closed circle, square, and diamond) conditions. The stressed phenotype was measured in the presence of both oligomycin and FCCP. Data represent mean ± SEM (n = 5). Student’s *t*-test: *P < 0.05, **P < 0.01, ***P < 0.001.

Next, we assessed mitochondrial biogenesis and oxygen consumption rate (OCR) in Capsaicin-treated ciBAs. The ratio of copy numbers of mitochondrial DNA to nuclear DNA was increased in ciBAs and further increased in Capsaicin-treated ciBAs (Fig. 2A). The elevated mitochondrial levels by Capsaicin were also supported by the increased expression of mitochondria-encoded genes, *MT-CYB* and *MT-ND5* (Fig. 2B). Immunoblotting analysis showed that mitochondrial marker proteins, COX4 and VDAC1, were more abundant in Capsaicin-treated ciBAs (Fig. 2C). The OCR in Capsaicin-treated ciBAs (RoFB + Capsaicin) was upregulated compared to that in the untreated ciBAs (RoFB) and typically varied by adding the inhibitors of the mitochondrial electron transfer chain (Fig. 2D). The OCR corresponding to basal respiration, maximal respiration, and spare respiratory capacity was significantly increased in Capsaicin-treated ciBAs (Fig. 2E). In particular, the OCR corresponding to ATP production and proton leak was also higher in Capsaicin-treated ciBAs than that in the untreated ciBAs (Fig. 2F). The energy phenotype profile displayed by a scatter plot of OCR and extracellular acidification rate (ECAR) indicated that Capsaicin-treated ciBAs had a higher metabolic potential under both basal and stressed conditions than the untreated ciBAs (Fig. 2G). These results suggested that treatment with Capsaicin activated mitochondrial respiration and glycolysis in ciBAs.

### Ligands for TRPV1 and TRPV4 regulate *UCP1* expression in ciBAs

**Figure 3:**
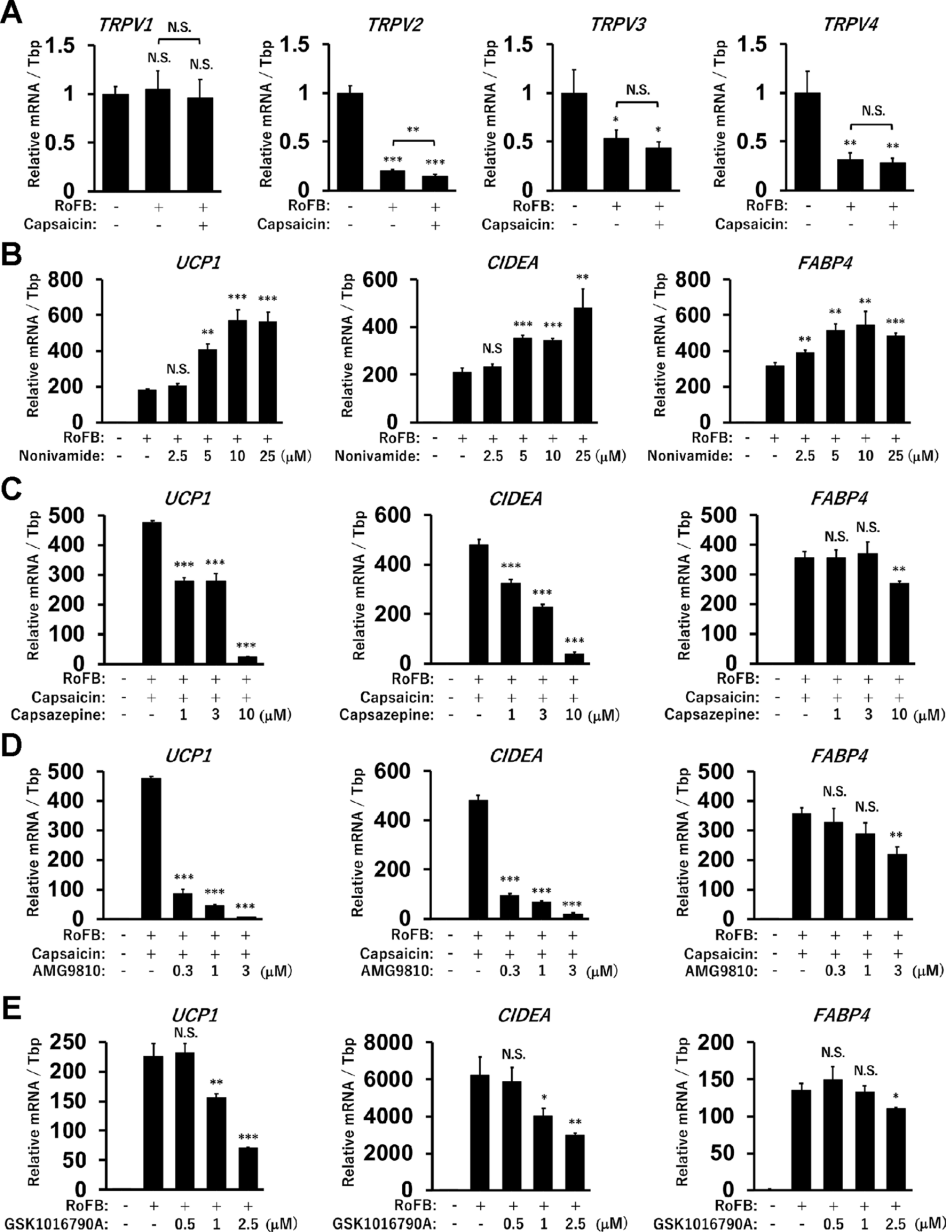
Effects of agonists and antagonists for TRPV1 and TRPV4 in ciBAs. (**A**) The expression of four TRP channels was quantified by qRT-PCR in the control, ciBAs, and Capsaicin-treated ciBAs. (**B–E**) The expression of *UCP1*, *CIDEA*, and *FABP4* was quantified in ciBAs induced in the presence of Nonivamide (**B**), Capsaicin and either Capsazepine (**C**) or AMG9810 (**D**), and GSK1016790A (**E**) at different concentrations as indicated. Data represent mean ± SD (n = 3). Student’s *t*-test: *P < 0.05, **P < 0.01, ***P < 0.001, N.S.; not significant.

To address whether TRPV1 regulates *UCP1* expression in ciBAs, agonists and antagonists of TRPV1 were examined. Thermos-sensitive TRP channels, TRPV1-4, are expressed in adipose tissues and are also involved in the regulation of brown adipocytes^38^. *TRPV1* expression did not change in either Capsaicin-treated or -untreated ciBAs, but the expression of the other TRP channels was decreased in these ciBAs (Fig. 3A). Treatment with Nonivamide, a TRPV1 agonist, activated the expression of *UCP1* and cell death-inducing DNA fragmentation factor-like effector A (*CIDEA*), a brown adipocyte-enriched gene, in a dose-dependent manner (Fig. 3B). *FABP4* expression was also increased by Nonivamide, similar to the results in the treatment with Capsaicin. On the contrary, Capsazepine and AMG9810, selective TRPV1 antagonists, repressed the transcriptional activation of *UCP1* and *CIDEA* genes by Capsaicin in a dose-dependent manner (Fig. 3C,D). However, Capsazepine at 10 μM and AMG9810 at 3 μM slightly inhibited *FABP4* expression, and the expression of both *UCP1* and *CIDEA* was more strongly repressed. TRPV4 has been reported to negatively regulate thermogenic gene expression in adipose tissues^39^. The treatment with GSK1016790A, a synthetic agonist of TRPV4, suppressed the expression of *UCP1* and *CIDEA* in a dose-dependent manner (Fig. 3E). These results indicated that the expression of *UCP1* and *CIDEA* genes was properly regulated through TRPV1 and TRPV4 in ciBAs.

### Capsaicin activates a broad range of metabolic genes in ciBAs

To identify which genes other than *UCP1* are regulated by Capsaicin treatment, genome-wide transcriptional analysis was performed. We have previously reported a comparison of RNA-sequencing (RNA-Seq) results between ciBAs and the control fibroblasts^27^. A comparison between the RNA-Seq data of Capsaicin-treated ciBAs (RoFB + Capsaicin) and the control fibroblasts (NoC) detected 1657 upregulated and 1279 downregulated differentially expressed genes (DEGs) (Supplementary Fig. S1A). Gene ontology (GO) enrichment analysis showed that the up-regulated DEGs were largely categorised into functional groups such as fatty acid metabolism and mitochondria, while the down-regulated DEGs were grouped into the extracellular matrix (Supplementary Fig. S1B,C), which is similar to our previous results on ciBAs and the control^27^.

**Figure 4:**
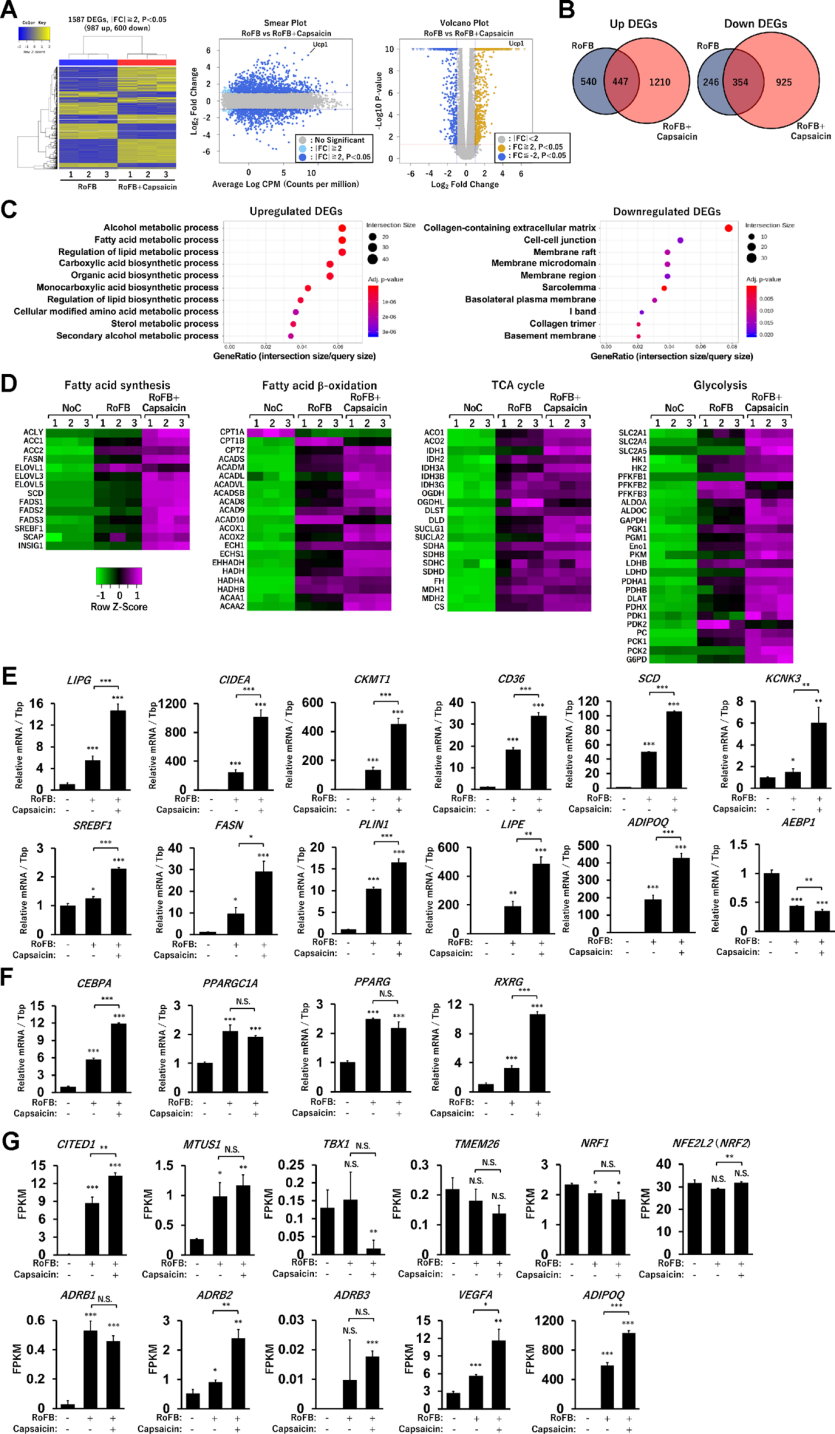
Genome-wide transcriptional analysis in Capsaicin-treated ciBAs. (**A**) Heat map and hierarchical clustering analysis represent 1587 differentially expressed genes (DEGs) (|fold change (FC)|≥ 2, P < 0.05) between ciBAs (RoFB) and Capsaicin-treated ciBAs (RoFB + Capsaicin). Smear and Volcano plots indicate logarithmic FC, P-value, and CPM (counts per million) between ciBAs and Capsaicin-treated ciBAs. (**B**) Venn diagrams represent overlap of up- and down-regulated DEGs between ciBAs and Capsaicin-treated ciBAs. (**C**) Gene ontology (GO) enrichment analysis was performed in the up- and down-regulated DEGs. Top 10 GO terms are represented. (**D**) Transcriptional profiles are shown as heat maps in functional groups such as fatty acid synthesis, β-oxidation, TCA cycle, and glycolysis pathway. The color scale shows z-scored fragments per kilobase of transcript per million mapped sequence reads (FPKM) representing mRNA levels of each gene in green (lower expression) and magenta (higher expression). (**E,F**) The expression of lipid metabolic genes (**E**) and transcription factors (**F**) was quantified by qRT-PCR analysis. (**G**) The FPKM values of brown/beige adipocyte-enriched genes such as *CITED1*, *MTUS1*, *TBX1*, *TMEM26*, *NRF1*, *NFE2L2* (*NRF2*), *ADBR1*, *ADBR2*, *ADBR3*, *VEGFA*, and *ADIPOQ* were obtained from the RNA-Seq results. Data represent mean ± SD (n = 3). Student’s *t*-test: *P < 0.05, **P < 0.01, ***P < 0.001, N.S.; not significant.

In the comparison between ciBAs (RoFB) and Capsaicin-treated ciBAs (RoFB + Capsaicin), 987 upregulated and 600 downregulated DEGs were identified (Fig. 4A). The smear and volcano plots showed that these DEGs with over two-fold changes (FCs) were appropriately distributed with widespread CPM (counts per million) and P-values. Venn diagrams represented that 447 up- and 354 down-regulated DEGs were overlapped in these ciBAs (Fig. 4B). The GO enrichment analysis suggested that the up- and down-regulated DEGs were mainly categorised into the functional groups related to lipid metabolism and extracellular matrix, respectively (Fig. 4C). Heat maps represented that the treatment with Capsaicin more enhanced the expression of a series of genes involved in the fatty acid synthesis, β-oxidation, tricarboxylic acid (TCA) cycle, glycolysis (Fig. 4D), and adaptive thermogenesis (Supplementary Fig. S2). Representative up- and down-regulated DEGs are listed in Supplementary Tables S1 and S2. The qRT-PCR analysis confirmed that Capsaicin-treated ciBAs increased the transcription of lipid metabolic genes such as *LIPG*, *CIDEA*, *CKMT1*, *CD36*, *SCD*, *KCNK3*, *SREBF1*, *FASN*, *PLIN1*, *LIPE*, and *ADIPOQ* (Fig. 4E). In contrast, the expression of *AEBP1*, a transcriptional repressor of adipogenesis, was more suppressed in Capsaicin-treated ciBAs. The RNA-Seq results also suggested that several transcription factors and coregulators for brown adipogenesis and *UCP1* transcription were increased by Capsaicin treatment (Supplementary Fig. S3A,B). Moreover, the qRT-PCR analysis confirmed that the expression of *CEBPA* and *RXRG*, but not *PPARGC1* and *PPARG*, was activated in Capsaicin-treated ciBAs (Fig. 4F). Several other nuclear receptors such as *LXRA*, *RORC*, *NR4A2*, and *NR4A3*, which might be involved in the transcriptional regulation of *UCP1* and other metabolic genes, were also activated (Supplementary Fig. S3C). In addition, the RNA-Seq results indicated the elevated expression of several brown/beige adipocyte-enriched genes (Fig. 4G). The expression of *CITED1* and *MTUS1* was upregulated in both ciBAs and Capsaicin-treated ciBAs, whereas the expression of *TBX1*, *TMEM26*, *NRF1*, and *NFE2L2* (*NRF2*) was not increased in ciBAs and Capsaicin-treated ciBAs. *PRDM16*, *FGF21*, and *CD137* genes were not detected owing to their low expression levels. Among β-adrenergic receptors, the expression of *ADBR2* was the highest, and its expression was enhanced by Capsaicin treatment. The RNA-Seq results also showed the elevated expression of *VEGFA* and *ADIPOQ*. These results indicated that Capsaicin activated the transcription of not only *UCP1* gene but also a broad range of metabolic genes.

### Reduced glycerol release and accumulation of triglycerides in Capsaicin-treated ciBAs

**Figure 5:**
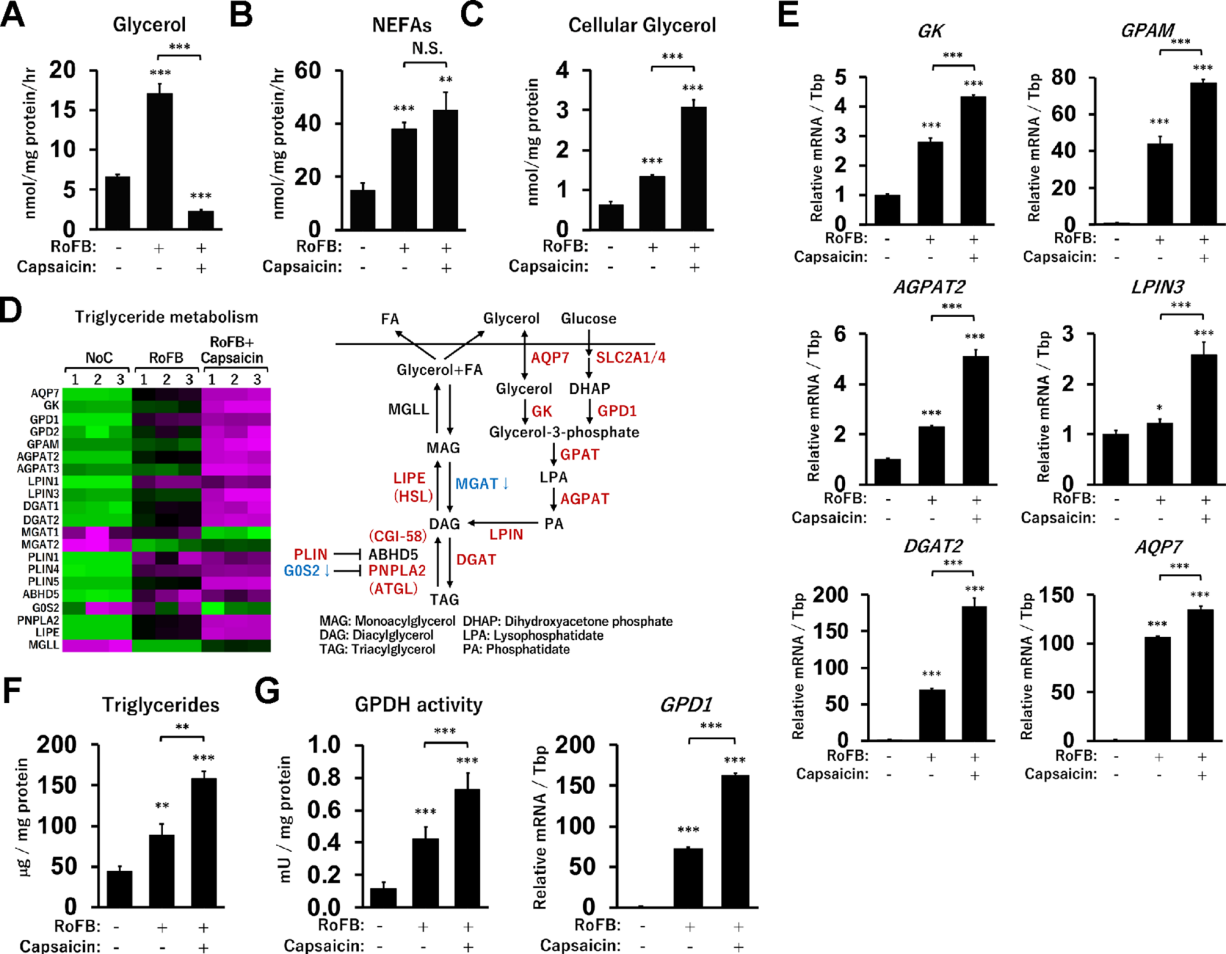
Glycerol and triglyceride metabolism in Capsaicin-treated ciBAs. (**A,B**) The secretion of glycerol (**A**) and non-esterified fatty acids (NEFAs) (**B**) into the culture medium was measured in the control, ciBAs, and Capsaicin-treated ciBAs. (**C**) Cellular glycerol levels were also measured. (**D**) A heat map represents the transcriptional changes of metabolic genes related to triglycerides metabolism. The color scale shows z-scored FPKM representing mRNA levels of each gene in green (lower expression) and magenta (higher expression). A schematic figure shows triglyceride metabolic pathway including up-regulated genes (highlighted by reddish-brown) and down-regulated genes (highlighted by blue) in Capsaicin-treated ciBAs. (**E**) The expression of *GK*, *GPAM*, *AGPAT2*, *LPIN3*, *DGAT2*, and *AQP7* was quantified by qRT-PCR analysis. (**F,G**) Cellular triglycerides (**F**) and GPDH activity (**G**) were measured in the control, ciBAs, and Capsaicin-treated ciBAs. *GPD1* mRNA was quantified by qRT-PCR. Data represent mean ± SD (n = 3). Student’s *t*-test: *P < 0.05, **P < 0.01, ***P < 0.001, N.S.; not significant.

Brown adipocytes possess distinct features of lipid metabolism that are different from those of white adipocytes^40,41^. Glycerol secretion was increased in ciBAs compared with that in the control, however, the secretion was apparently reduced in Capsaicin-treated ciBAs (Fig. 5A). Reduced glycerol release was also observed a few days after the treatment with Capsaicin (Supplementary Fig. S4). Secreted non-esterified fatty acids (NEFAs) were not altered between Capsaicin-treated and untreated ciBAs (Fig. 5B), indicating that lipolysis was not reduced in Capsaicin-treated ciBAs. In contrast, cellular glycerol content was higher in Capsaicin-treated ciBAs than in the untreated ciBAs (Fig. 5C). These observations indicated that glycerol secreted into the medium was actively recovered in Capsaicin-treated ciBAs. A heat map obtained from the RNA-Seq results showed that a series of genes encoding enzymes involved in triglyceride metabolism were activated in Capsaicin-treated ciBAs (Fig. 5D). Reportedly, recovered glycerol is first phosphorylated by glycerol kinase (GK), then, recycled into triglycerides by GAPT, AGPAT, LPIN, and DGAT enzymes^42^. The qRT-PCR analysis confirmed that the expression of *GK*, *GPAM*, *AGPAT2*, *LPIN3*, *DGAT2*, and *AQP7* was increased in Capsaicin-treated ciBAs (Fig. 5E). Moreover, cellular triglycerides were more accumulated in Capsaicin-treated ciBAs than in untreated ciBAs (Fig. 5F). The triglyceride accumulation was also supported by higher glycerol-3-phosphate dehydrogenase (GPDH) activity and increased expression of *GPD1* gene, responsible for cytoplasmic GPDH activity (Fig. 5G). Taken together, these results suggested that Capsaicin could promote glycerol recycling in ciBAs through the transcriptional activation of related metabolic genes.

### Capsaicin negatively affects adipocyte browning in mesenchymal stem cell-derived adipocytes

**Figure 6:**
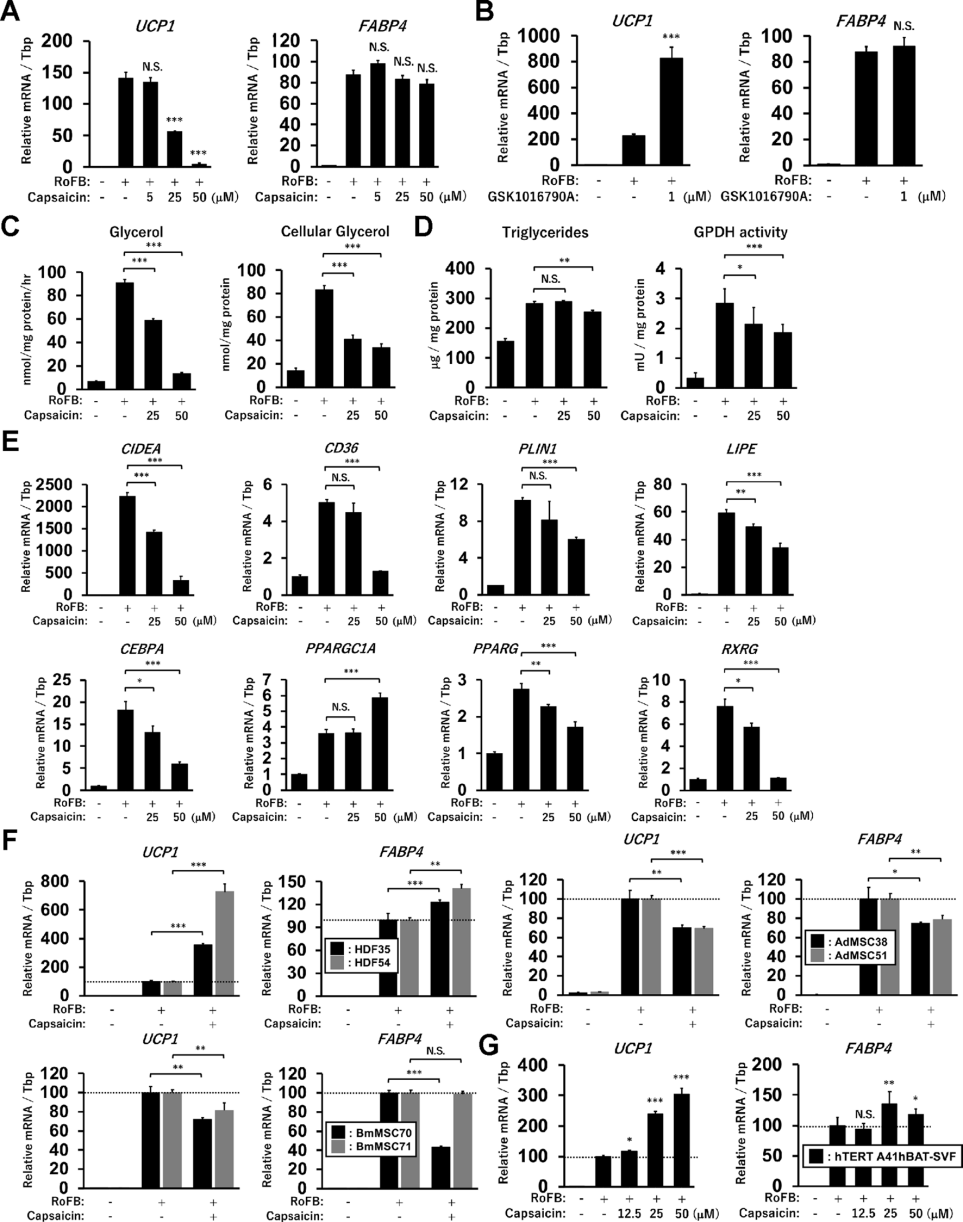
Effects of Capsaicin in adipose tissue-derived mesenchymal stem cell (AdMSC)-derived adipocytes. (**A**) The expression of *UCP1* and *FABP4* was quantified in AdMSC-derived adipocytes differentiated in the presence of Capsaicin at different concentrations as indicated. (**B**) The qRT-PCR analysis was performed in the adipocytes differentiated in the presence of GSK1016790A. (**C**) Glycerol secretion and cellular glycerol levels were quantified in AdMSC-derived adipocytes treated with Capsaicin at 25 μM and 50 μM. (**D**) Triglyceride content and GPDH activity were measured. (**E**) The expression of metabolic genes and transcription factors analysed in ciBAs was quantified. (**F**) Expression change of *UCP1* and *FABP4* by Capsaicin treatment at 25 μM was evaluated in ciBAs derived from two different HDFs (HDF35 and HDF54) and in the adipocytes derived from AdMSCs (AdMSC38 and AdMSC51) and BmMSCs (BmMSC70 and BmMSC71). (**G**) The expression was also measured in adipocytes differentiated from an immortalised human brown preadipocyte cell line (hTERT A41hBAT-SVF). Data represent mean ± SD (n = 3). Student’s *t*-test: *P < 0.05, **P < 0.01, ***P < 0.001, N.S.; not significant.

We have previously reported that adipocytes differentiated from AdMSCs under the same culture conditions as ciBAs predominantly expressed *UCP2* rather than *UCP1*^27^. To verify whether Capsaicin treatment similarly induces *UCP1* expression, AdMSC-derived adipocytes were tested. The treatment with Capsaicin reduced the expression of *UCP1* in a dose-dependent manner without a significant change in *FABP4* expression (Fig. 6A). In contrast, the treatment with GSK1016970A upregulated *UCP1* expression (Fig. 6B). These responses against TRPV1 and TRPV4 agonists in AdMSC-derived adipocytes were opposite to those in ciBAs. Although glycerol secretion was reduced by Capsaicin in AdMSC-derived adipocytes, cellular glycerol content was also reduced (Fig. 6C). In addition, triglyceride accumulation and GPDH activity were reduced by Capsaicin treatment (Fig. 6D). The adipocytes decreased the expression of several metabolic genes such as *CIDEA*, *CD36*, *PLIN1*, and *LIPE*, which was activated by Capsaicin in ciBAs (Fig. 6E). In particular, reduced expression of key transcriptional factors such as *CEBPA*, *PPARG*, and *RXRG* suggested that Capsaicin treatment had adverse effects on lipid metabolism and metabolic gene expression in AdMSC-derived adipocytes. To further confirm the effects of Capsaicin on the regulation of *UCP1* expression, two other lines of human dermal fibroblasts (HDFs), AdMSCs, and bone marrow-derived mesenchymal stem cells (BmMSCs) were examined (Fig. 6F). ciBAs converted from HDF35 and HDF54 in the presence of Capsaicin exhibited higher expression levels of both *UCP1* and *FABP4* genes. In contrast, *UCP1* expression was either decreased or showed almost no change in MSC-derived adipocytes differentiated in the presence of Capsaicin from the two AdMSCs (AdMSC38 and AdMSC51) and BmMSCs (BmMSC70 and BmMSC71). On the contrary, Capsaicin activated the expression of *UCP1* and *FABP4* in adipocytes differentiated from an immortalised human brown preadipocyte cell line (hTERT A41hBAT-SVF) (Fig. 6G). Collectively, these results suggested that Capsaicin positively regulated *UCP1* expression in ciBAs and the immortalised human brown adipocytes but not in MSC-derived adipocytes.

## Discussion

**Figure 7:**
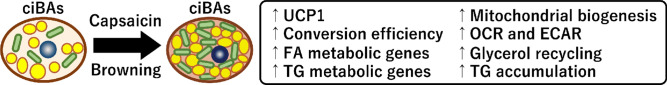
Schematic presentation of the browning effects promoted by Capsaicin in ciBAs. The treatment with Capsaicin induces the expression of the brown adipocyte-specific gene, *UCP1*, and a series of metabolic genes involved in fatty acid (FA) metabolism, triglyceride (TG) metabolism, glycolysis pathway, and adaptive thermogenesis. Moreover, Capsaicin increases the efficiency of the direct conversion, mitochondrial biogenesis, OCR and ECAR, glycerol recycling, and TG accumulation, which are closely associated with functions in brown adipocytes.

In this study we successfully identified the direct effects of Capsaicin on adipocyte browning in ciBAs converted from human dermal fibroblasts (Fig. 7). The treatment of Capsaicin during the direct conversion induced *UCP1* expression and promoted the conversion efficiency itself. The effects of TRPV1 agonists and antagonists on *UCP1* expression implicated that the effects could be mediated through TRPV1 in ciBAs. The RNA-Seq analysis revealed that Capsaicin activated not only *UCP1* gene but also a series of metabolic genes involved in triglyceride metabolism, fatty acid metabolism, glycolysis pathway, and adaptive thermogenesis. Many of them were overlapped with genes activated during the direct conversion into ciBAs, as shown in our previous analysis^36^, indicating that these metabolic genes are closely associated with functions in brown adipocytes. In addition, this study revealed that Capsaicin activated mitochondrial biogenesis, oxygen consumption, glycolysis, glycerol recycling, and triglyceride accumulation. In this study, the direct effects of Capsaicin on ciBAs were observed as a result of both the enhanced populations of ciBAs and their maturation. The increased conversion efficiency likely contributes to the metabolic gene expression and the observations unique to brown adipocytes. These brown-like phenotypes activated by Capsaicin in ciBAs are likely involved in the process of their maturation and adipocyte browning. Overall, prolonged treatment with Capsaicin directly promotes the conversion efficiency and adipocyte browning in ciBAs.

Our previous report suggested that the direct conversion to ciBAs underwent genome-wide transcriptional changes similar to the adipocyte browning in AdMSCs^36^. In contrast, one of the most characteristic differences between them was the expression pattern of major uncoupling proteins. ciBAs more expressed higher levels of *UCP1*, whereas the AdMSC-derived adipocytes predominantly expressed *UCP2*. The latter possesses a weaker uncoupling activity and is expressed in WAT rather than BAT. To further clarify the differences between these adipocyte models, in this study, we examined the effects of Capsaicin in AdMSC-derived adipocytes differentiated under the same culture conditions as ciBAs. Notably, Capsaicin rather repressed the expression of *UCP1* and several metabolic genes in AdMSC-derived adipocytes (Fig. 6A,E). In contrast, the TRPV4 agonist, GSK1016790A, increased *UCP1* expression (Fig. 6B), which was opposite to the response in ciBAs. In addition, Capsaicin did not similarly regulate the cellular levels of glycerol and triglycerides (Fig. 6C,D). Other reports have indicated that in 3T3-L1 white adipocytes Capsaicin inhibited adipogenesis, triglyceride accumulation, GPDH activity, and the expression of *PPARG* and *CEBPA*^28,43^, suggesting that TRPV1 might play a different role in white and brown adipocytes. Furthermore, the effect of Capsaicin was validated using an immortalised brown preadipocyte cell line isolated from human neck fat that activated *UCP1* expression in response to Capsaicin (Fig. 6G). Consistent with our findings, several studies in mice have shown that TRPV1 and TRPV4 positively and negatively regulate thermogenic genes and brown phenotypes^32,38,39^. These findings supported that ciBAs reflected more appropriate responses to these agonists than AdMSC-derived adipocytes, at least under the culture conditions.

It has been known that secreted glycerol is not recycled in white adipocytes due to a lower activity of glycerol kinase^44^. One of the characteristic metabolic changes induced by Capsaicin was the apparent reduction in glycerol release (Fig. 5A). In general, it is considered to be caused by either decreased lipolysis or increased glycerol uptake. However, it was unlikely that lipolysis was reduced because the level of NEFA secretion did not much change significantly in Capsaicin-treated ciBAs (Fig. 5B). This was also supported by the increase of the OCR and ECAR in Capsaicin-treated ciBAs (Fig. 2G), which indicated that energy expenditure was more enhanced by Capsaicin in ciBAs. In addition, the activation of *GK*, *GPAM*, *AGPAT2*, *LPIN3*, and *DGAT2* genes encoding key enzymes in the triglyceride synthesis pathway likely contributed to glycerol recycling^41,42^ (Fig. 5E). The triglyceride accumulation in Capsaicin-treated ciBAs was also supported by elevated GPDH activity along with *GDP1* expression (Fig. 5G). It should also be noted that glycerol-3 phosphate (G3P) produced by glycerol kinase from glycerol is a key component for the NADH-G3P shuttle^4^. This shuttle allows for rapid aerobic ATP synthesis in the mitochondria, which contributes to adaptive thermogenesis in brown adipocytes^45^. Taken together, ciBAs could elicit Capsaicin-induced metabolic changes unique to brown adipocytes.

Capsaicin treatment upregulated several transcription factors and cofactors that directly regulate *UCP1* transcription in ciBAs (Fig. 4F, Supplementary Fig. S3). Reportedly, CEBPA directly and indirectly regulates *UCP1* transcription and adipocyte differentiation^46^. Moreover, several nuclear receptors are involved in the direct regulation of *UCP1* in response to steroid hormones, retinoids, and lipid metabolites^23,47^. In this study, the RNA-Seq data and qRT-PCR results demonstrated that *RXRG, LXRA*, *RORC*, *NR4A2*, and *NR4A3* were upregulated by Capsaicin. RXRG is especially important for brown adipogenesis by forming heterodimers with other nuclear receptors such as PPARs, RARs, LXRs, and TRs to activate their target genes including *UCP1*^48^. Although cellular metabolic changes induced by Capsaicin might affect its expression, *UCP1* transcription could be at least partially activated by CEBPA, RXRG, and several nuclear receptors whose expression was activated by Capsaicin. However, further studies are required to reveal the precise molecular mechanism underlying the activation of *UCP1* gene by the treatment with Capsaicin. Of note, Capsaicin treatment enhanced *ADRB2* transcription at a higher level than the other *ADRBs* (Fig. 4G). ADRB2 is known to be the main β-adrenergic receptor functional in human brown adipocytes^49^. In addition, the activation of *VEGFA* expression by Capsaicin might be involved in the process of vascularisation during adipocyte browning *in vivo*^10^.

The manipulation of brown adipocyte population in our body by small molecules has a therapeutic potential for the treatment of obesity-associated metabolic diseases including type 2 diabetes mellitus and cardiovascular diseases. However, it is not feasible to isolate a sufficient amount of homogeneous human brown adipocytes every time the effects of bioactive molecules are evaluated^37^. In our previous studies, we developed a technique for serum- and transgene-free direct conversion of human dermal fibroblasts into brown adipocytes^33,34^. This method enables convenient and reproducible preparation for human brown adipocyte-like cells applicable to basic, pharmacological, and clinical research^35^. In this study, we demonstrated a successful evaluation of the direct effects of Capsaicin on adipocyte browning through TRPV1 in ciBAs. In conclusion, ciBAs could serve as a promising model especially for the first screening of bioactive molecules and dietary compounds with a potential ability to directly promote adipocyte browning and brown adipogenesis. In other words, ciBAs could assist in the characterisation of dietary factors and offer novel insights into the nutritional intervention strategies aimed at preventing and managing obesity and related metabolic diseases.

## Methods

### Cell culture

Fibroblasts derived from a human subject aged 38 years (HDF38) were mainly used^36^. Approximately 1.5 × 10^5^ cells were seeded on a 35-mm dish with high-glucose Dulbecco's Modified Eagle Medium (DMEM) (11995–065, Gibco, MA, USA) supplemented with 10% fetal bovine serum (FBS) (HyClone, UT, USA) and penicillin/streptomycin (Gibco). After reaching 80–90% confluence, the medium was changed to start the direct conversion into ciBAs with the serum-free brown adipogenic medium (SFBAM) prepared from high-glucose DMEM (11995–065, Gibco) supplemented with linoleic acid- and oleic acid-albumin (L9655-5ML, Sigma-Aldrich, MO, USA), 3,3’,5 triiodothyronine (T3) (Sigma-Aldrich), dexamethasone (FUJIFILM Wako, Osaka, Japan), 3-isobutyl-1-methylxanthine (IBMX) (FUJIFILM Wako), human recombinant insulin (FUJIFILM Wako), L-ascorbic acid-2-phosphate (Sigma-Aldrich), and penicillin/streptomycin (Gibco). As reported previously^34^, the human fibroblasts were incubated with SFBAM either with or without the chemical combination RoFB, which consists of 1 μM Rosiglitazone (FUJIFILM Wako), 7.5 μM Forskolin (FUJIFILM Wako), and 20 ng/ml human recombinant BMP7 (FUJIFILM Wako) for 3 weeks unless otherwise indicated. Capsaicin (030–11353, FUJIFILM Wako) was added in combination with RoFB at a concentration of 25 μM unless otherwise indicated. The medium was changed every 3 days. Other TRPV1 and TRPV4 ligands, Nonivamide (sc-202735, Santa Cruz Biotechnology, CA, USA), Capsazepine (037–23171, FUJIFILM Wako), AMG9810 (015–25071, FUJIFILM Wako), and GSK1016790A (G0798, Sigma-Aldrich) were used at different concentrations as indicated. Other lines of human dermal fibroblasts (HDF35 and HDF54) were purchased from DS Pharma Biomedical Co. (Osaka, Japan)^36^. The immortalised preadipocyte cell line isolated from human deep neck fat tissue (hTERT A41hBAT-SVF) was purchased from the American Type Culture Collection (CRL-3385, ATCC, VA, USA). They were maintained and differentiated in the same manner as described above.

MSCs derived from the adipose tissue of human subjects at age 46 (AdMSC46), 38 (AdMSC38), and 51 (AdMSC51) were purchased from TaKaRa Bio (C-12977, TaKaRa Bio, Shiga, Japan) and cultured in Mesenchymal Stem Cell Growth Medium 2 (TaKaRa Bio)^36^. After reaching 80–90% confluence, AdMSCs were differentiated into mature adipocytes with SFBAM and the chemical cocktail, RoFB, in either the presence or absence of Capsaicin as described above. MSCs derived from the bone marrow of human subjects aged 70 (BmMSC70) and 71 (BmMSC71) were also purchased from TaKaRa Bio (C-12974, TaKaRa Bio). All the commercial human cells have been approved for in vitro research use only. All experimental procedures for cell cultures were conducted in accordance with the general guidelines in Kyoto Prefectural University of Medicine.

### Quantitative real-time PCR (qRT-PCR)

Total RNA was extracted from the control fibroblasts, ciBAs, and AdMSCs cultured in each experimental condition using FastGene RNA Basic Kit (Nippon Genetics, Tokyo, Japan). Reverse transcription was conducted using ReverTra Ace qPCR RT Master Mix with gDNA Remover (TOYOBO, Osaka, Japan). The qRT-PCR analysis was performed using Power SYBR Green PCR Master Mix (Applied Biosystems, MA, USA). The reactions were carried out in triplicate and under the following conditions: 10 min at 95 °C, followed by 40 cycles of 15 s at 95 °C and 60 s at 60 °C. All the results were normalised to *TBP* mRNA levels. The ratio of *UCP1* to *FABP4* mRNA was calculated as an indicator for adipocyte browning. The primer sequences used for qRT-PCR are listed in Supplementary Table S3. Unless otherwise indicated, the average of the three biological replicates was calculated.

### Quantification of mitochondrial DNA

Total genomic DNA was extracted with NucleoSpin Tissue (TaKaRa Bio) from the control fibroblasts and ciBAs treated either with or without Capsaicin. mtDNA copy numbers were measured by qPCR using 10 ng of total genomic DNA and Power SYBR Green PCR Master Mix. Each mtDNA was normalised to corresponding nuclear DNA level. Primer sequences for the quantification of mitochondrial and nuclear DNA were as follows: mtDNA-Fwd, ACACCCTCCTAGCCTTACTAC; mtDNA-Rev, GATATAGGGTCGAAGCCGC; nuDNA-Fwd, AGGGTATCTGGGCTCTGG; NuDNA-Rev, GGCTGAAAAGCTCCCGATTAT^34^. The average of the three biological replicates was calculated.

### Immunoblotting

For immunoblot analysis, total proteins were extracted from the control fibroblasts, ciBAs, and Capsaicin-treated ciBAs with RIPA buffer (FUJIFILM Wako) including phosphatase inhibitor cocktail (FUJIFILM Wako) and protease inhibitor cocktail (FUJIFILM Wako). The extracted proteins were subjected to sodium dodecyl sulfate–polyacrylamide gel electrophoresis using a 10% gel concentration and transferred to a polyvinylidene fluoride membrane (Thermo Fisher Scientific, MA, USA). The membranes were blocked with 3% skim milk followed by incubation with antibodies against UCP1 (MAB6158, R&D Systems, MN, USA), COX4 (#4850, Cell Signalling Technology, MA, USA), VDAC1 (ab14734, Abcam, Cambridge, UK), or β-Actin (A5316, Sigma-Aldrich) at 4 ˚C overnight. The membranes were incubated with either HRP-conjugated anti-rabbit or anti-mouse secondary antibodies (Santa Cruz Biotechnology, CA, USA) for 1 h at room temperature. Immunoreactive bands were detected by Immobilon Western Chemiluminescent HRP Substrate (Merck Millipore, Darmstadt, Germany). The intensity of each band was quantified by densitometry using ImageJ software (National Institutes of Health). The experiments were performed independently at least twice.

### Immunocytochemistry

The cells were incubated with 1 µM Lipi-Red (Dojindo, Kumamoto, Japan) for 30 min at 37 °C in 5% CO_2_, according to the manufacturer’s instructions. Then the cells were fixed with 4% paraformaldehyde for 10 min. After washing with phosphate-buffered saline (PBS), the cells were incubated with PBS containing 0.1% Triton X-100 for 5 min. After incubation, they were blocked with PBS containing 3% skim milk for 1 h at room temperature and incubated again with UCP1 antibody (ab10983, Abcam) at 1/1000 dilution overnight at 4 °C. After washing with PBS, the cells were incubated with Alexa Fluor 488 donkey anti-rabbit IgG (Invitrogen, CA, USA) for 1 h at room temperature. Subsequently, the cell nuclei were stained with 4′,6-diamidino-2-phenylindole (DAPI) solution (Dojindo). All images were obtained using a BZ-X710-All-in-One Fluorescence Microscope (Keyence, Osaka, Japan) with a 20X objective lens (CFI Plan Fluor 20X, Nikon, Tokyo, Japan). All the scale bars represent 100 μm. To evaluate the direct conversion efficiency, the number of DAPI-positive cells, Lipi-Red positive cells, and UCP1-positive cells were counted from at least three different optical sections. The range of the number of cells was from 110 to 200 in each optical section. The experiments were performed independently at least twice.

### Measurement of OCR

For measurement of OCR by mitochondria, human dermal fibroblasts were seeded on a 96-well plate and converted to ciBAs by either RoFB or RoFB + Capsaicin in SFBAM for 3 weeks. As a control, the fibroblasts were cultured in parallel with the medium. Before the measurement, the cells were washed and incubated with non-buffered DMEM supplemented with 25 mM glucose, 2 mM glutamine, and 1 mM pyruvate at 37 °C in a non-CO_2_ incubator for 1 h. Then, OCR was measured by the Seahorse XF96 Extracellular Flux Analyzer (Seahorse Bioscience Inc., MA, USA) according to the manufacturer’s instructions. During the analysis, oligomycin, FCCP, and antimycin A/rotenone were added into each well via an injection apparatus to final concentrations at 2 μM, 0.3 μM, and 0.5 μM, respectively. Extracellular acidification rate (ECAR) was simultaneously measured. The experiments were performed twice independently.

### Cell viability and cytotoxicity assays

Cell viability was measured by using Cell Counting Kit-8 (Dojindo), according to the manufacturer’s instructions. The human fibroblasts were seeded on a 96 well plate and ciBAs were induced for 3 weeks either with or without Capsaicin as indicated. The cell viability was presented as a percentage of the control cells cultured in the medium only in parallel. To assess cytotoxicity, the activity of lactate dehydrogenase (LDH) released into the culture supernatants was measured by Cytotoxicity LDH Assay Kit-WST (Dojindo) according to the manufacturer’s instructions. The average of three biological replicates was calculated.

### Measurement of glycerol, NEFAs, triglycerides, and GPDH activity

Cell culture supernatants and extracted cell lysates were collected from the control fibroblasts and Capsaicin-treated and -untreated ciBAs. The amount of free glycerol and non-esterified fatty acids (NEFAs) was measured by Free Glycerol Assay Kit (ab65337, Abcam) and Free Fatty Acid Assay Kit (ab65341, Abcam), respectively, according to the manufacturer’s instructions. For measurement of cellular triglyceride content, lipids were extracted from the control fibroblasts and ciBAs by Folch method. Triglycerides in each extract were determined by LabAssay Triglyceride kit (FUJIFILM Wako). In addition, glycerol-3-phosphate dehydrogenase (GPDH) activity was measured in cell lysates extracted from the control fibroblasts and ciBAs by GPDH Assay kit (AK01, Cosmo Bio Co., Ltd., Tokyo, Japan) according to the manufacturer’s instructions. All the experiments were performed in triplicates. The levels of glycerol, NEFAs, triglycerides, and GPDH activity were normalised to protein amounts in each cell culture. The experiments were performed independently at least twice.

### RNA-Sequencing (RNA-Seq)

RNA-Seq results in the control fibroblasts (NoC) and ciBAs (RoFB) have been previously reported^36^. In this study, total RNA was prepared from Capsaicin-treated ciBAs (RoFB + Capsaicin) derived from HDF38 by FastGene RNA Premium Kit (Nippon Genetics, Tokyo, Japan). RNA integrity number (RIN) values were over 9 in all the RNA samples. The library was prepared by TruSeq stranded mRNA LT Sample Prep Kit (Illumina, CA, USA), following the manufacturer’s low sample (LS) protocol. A 100 bp paired-end sequencing was performed by NovaSeq 6000 System (Illumina). Trimmed reads were mapped to a reference genome (NCBI GRCh37) with HISAT2. After the read mapping, StringTie was used for transcript assembly. After the assembly, the abundance of gene/transcript was calculated from the read counts and normalised as fragments per kilobase of transcript per million mapped sequence reads (FPKM). For identification of DEGs, statistical analysis was performed by FC and exact test using edgeR per comparison pair. Significant results satisfying the conditions of |FC|≥ 2 and the exact test *p* value < 0.05 were selected. If more than one read count value was zero, it was not included in the analysis.

### Data analysis

Heat maps were generated using Heatmapper (http://www.heatmapper.ca/)^50^. Hierarchical clustering analysis was based on Euclidean distance. Each row represents a gene, and each column represents z-scored FPKM of each sample. The green and magenta gradients represent lower and higher gene expression, respectively. Gene ontology (GO) enrichment analysis was performed by DAVID Bioinformatics Resources 6.8 (https://david.ncifcrf.gov/)^51^.

### Statistical Analyses

All the results are presented as the mean ± SD or SEM. Statistical analyses were performed by a two-tailed Student’s *t*-test in the Excel (Microsoft) program. Statistical significance was defined as *p* < 0.05.

## Supplementary Information

Supplementary Information.

## Data Availability

The datasets generated or analysed in the present study are available from the corresponding authors upon reasonable request.
