# Supplementary material for: Capsaicin directly promotes adipocyte browning in the chemical compound-induced brown adipocytes converted from human dermal fibroblasts
Source: Sci Rep. 2022 Apr 22;12:6612. doi: 10.1038/s41598-022-10644-8 (PMC9033854; doi:10.1038/s41598-022-10644-8)
Supplement: Supplementary file 1 — Supplementary Information. [file 41598_2022_10644_MOESM1_ESM.pdf]

Capsaicin directly promotes adipocyte browning in the chemical compound-induced brown adipocytes converted from human dermal fibroblasts

Yukimasa Takeda and Ping Dai

Department of Cellular Regenerative Medicine, Graduate School of Medical Science, Kyoto Prefectural University of Medicine, 465 Kajii-cho, Kawaramachi-Hirokoji, Kamigyo-ku, Kyoto 602-8566, Japan

Supplementary Information

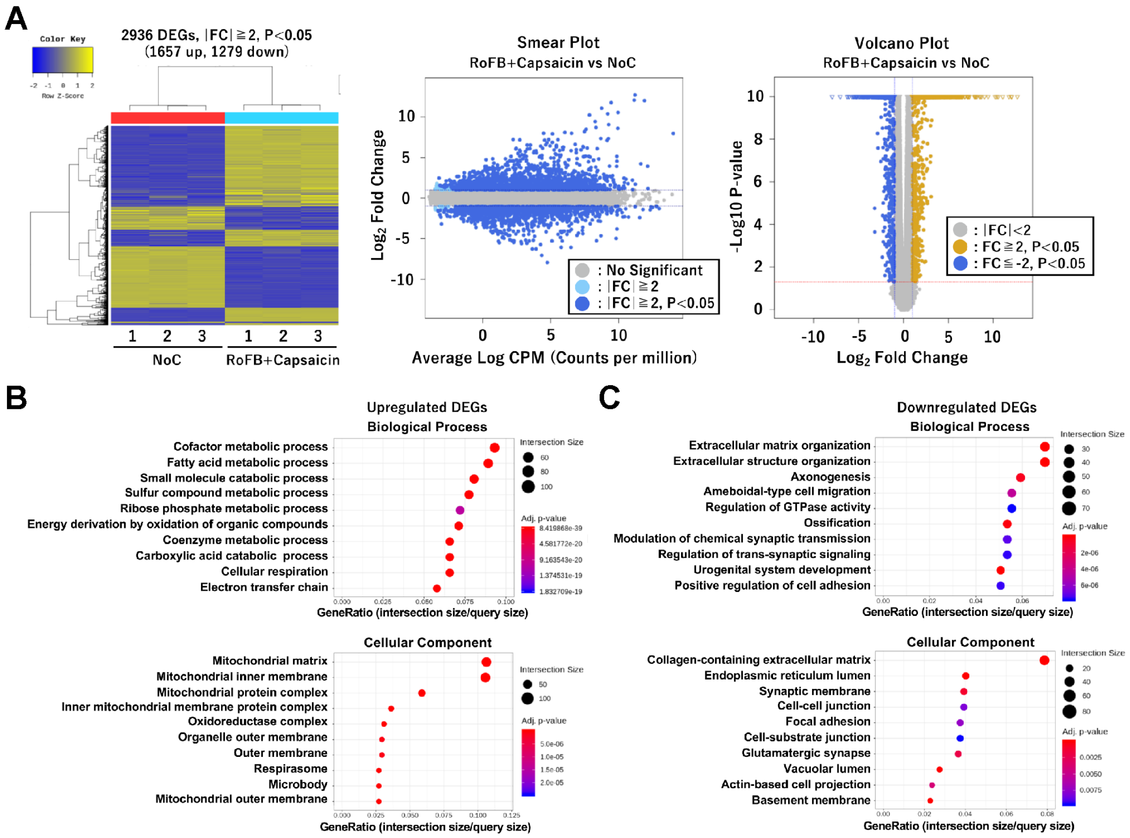

Supplementary Figure S1. Comparison of genome-wide transcriptional analysis between Capsaicin-treated ciBAs (RoFB+Capsaicin) and the control fibroblasts (NoC) cultured in the SFBAM only in parallel. (**A**) Heat map and hierarchical clustering analysis represent 2936 differentially expressed genes (DEGs) (|fold change (FC)|≥2, P<0.05) between NoC and RoFB+Capsaicin. Smear and Volcano plots indicate logarithmic fold change, P-value, and CPM (counts per million) between them. (**B,C**) Gene ontology (GO) enrichment analysis was performed in the 1657 up- and 1279 down-regulated DEGs. Top 10 GO terms are represented in the category of Biological Process and Cellular Component.

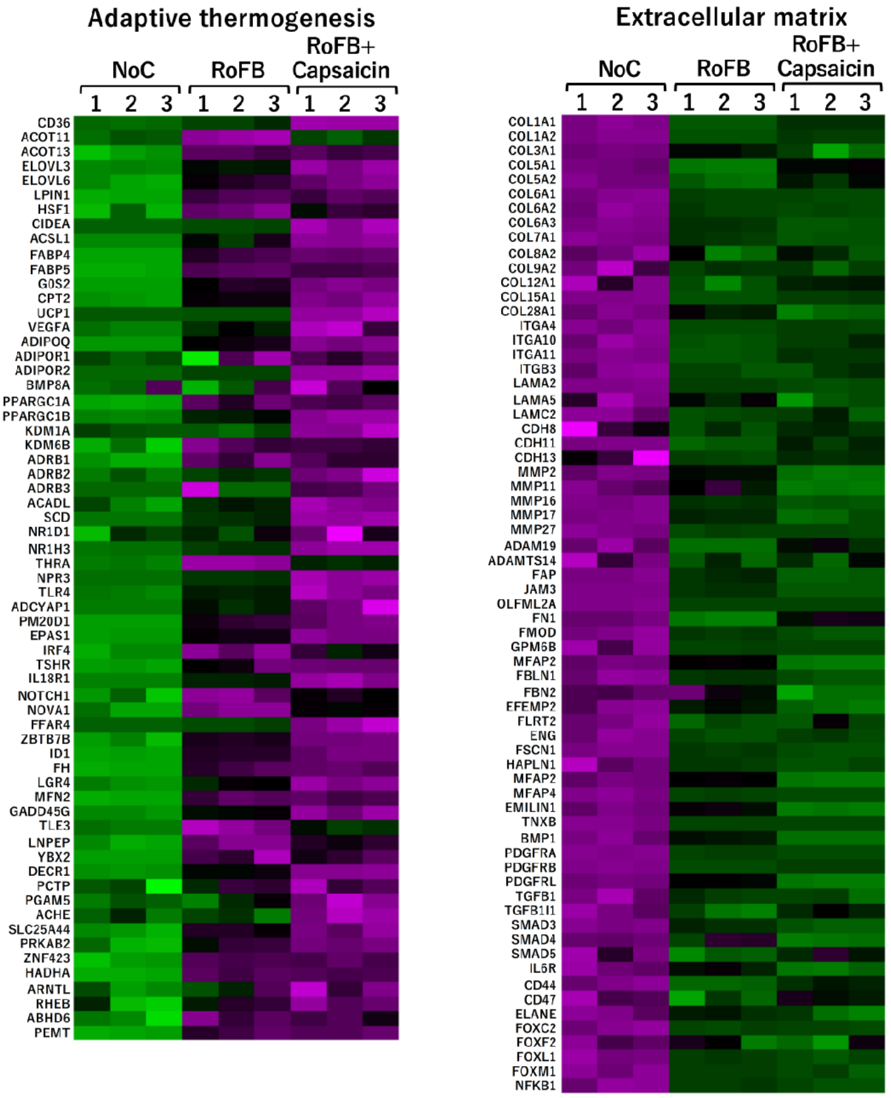

Supplementary Figure S2. Heat maps represent transcriptional profiles of functional genes related to adaptive thermogenesis and extracellular matrix. The color scale shows z-scored fragments per kilobase of transcript per million mapped sequence reads (FPKM) representing mRNA levels of each gene in green (lower expression) and magenta (higher expression).

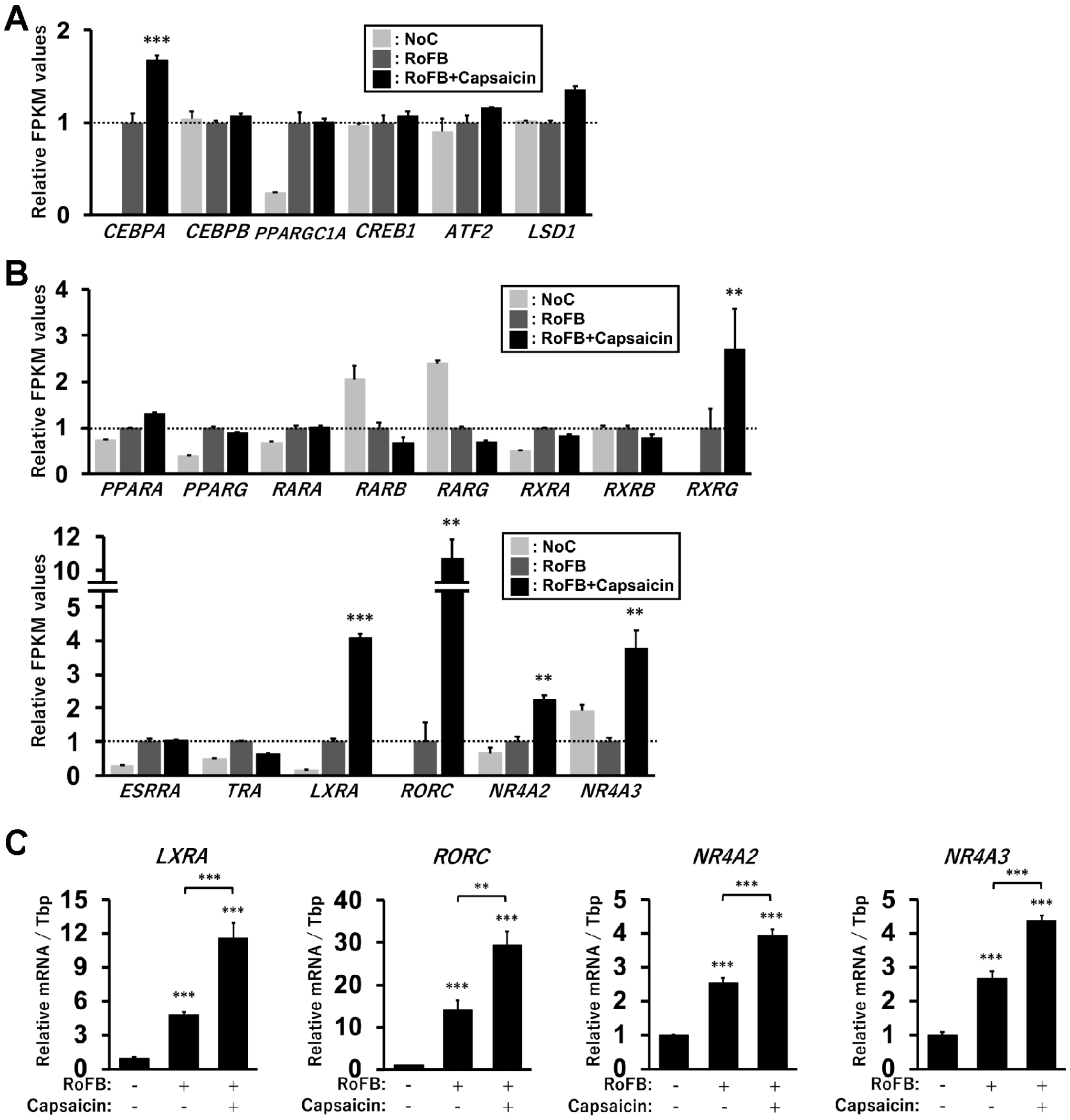

Supplementary Figure S3. The expression of transcription factors and nuclear receptors in Capsaicin-treated ciBAs. (**A,B**) Relative FPKM values obtained from the RNA-Seq results were shown in transcription factors (**A**) and nuclear receptors (**B**) which are involved in the regulation of *UCP1* and metabolic gene expression. (**C**) The expression of *LXRA*, *RORC*, *NR4A2*, and *NR4A3* was quantified by qRT-PCR analysis in the control, ciBAs, and Capsaicin-treated ciBAs. Data represent mean±SD (n=3). Student's t-test: **P<0.01, ***P<0.001.

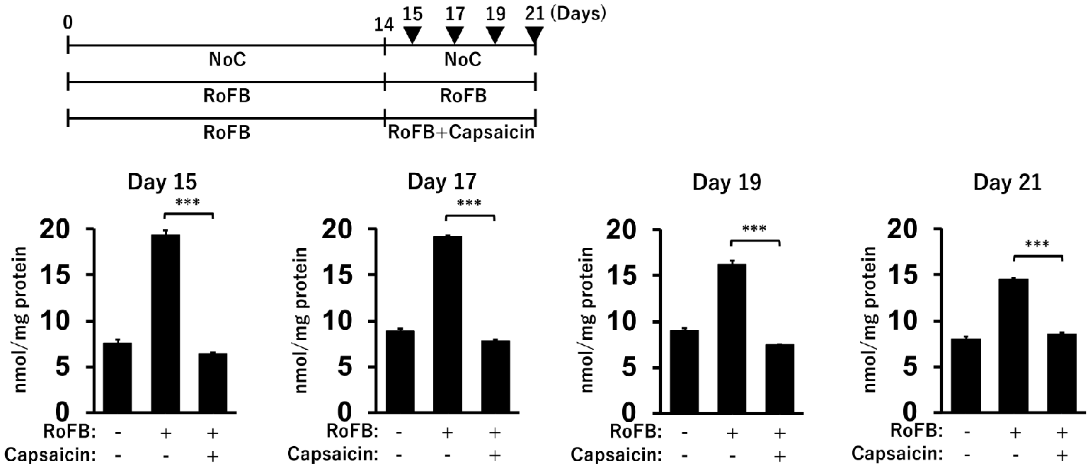

Supplementary Figure S4. Glycerol secretion after the treatment with Capsaicin according to an experimental scheme. The secretion of glycerol into the culture medium was measured at Day 15, Day 17, Day 19, and Day 21 in the control cells (NoC), ciBAs (RoFB), and ciBAs treated with Capsaicin (RoFB+Capsaicin) from Day 14. Data represent mean±SD. Student's t-test: ***P<0.001.

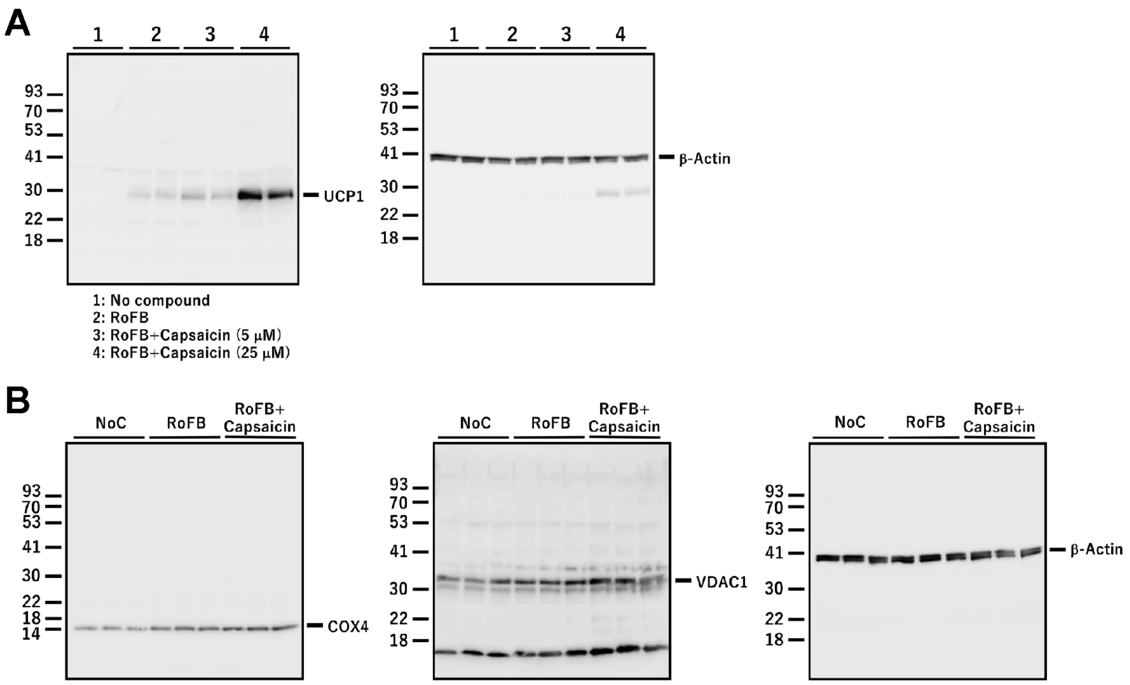

Supplementary Figure S5. Images of full-length western blot. (**A,B**) The raw results of western blot analysis are shown for Figure 1D (**A**) and Figure 2C (**B**).

Supplementary Table S1. List of representative metabolic genes upregulated by Capsaicin in ciBAs.

Gene name	Description	Fold Change	Adjusted p-value
UCP1	Uncoupling protein 1	43.71	0.00E+00
PLA2G2A	Phospholipase A2 group IIA	30.21	0.00E+00
ABCG1	ATP binding cassette subfamily G member 1	21.95	1.60E-207
LIPG	Lipase G, endothelial type	20.84	8.50E-100
SLC2A5	Solute carrier family 2 member 5	16.28	5.39E-136
MOGAT1	Monoacylglycerol O-acyltransferase 1	14.74	1.47E-62
CIDEA	Cell death inducing DFFA like effector a	10.81	2.78E-189
FFAR4	Free fatty acid receptor 4	10.18	4.19E-44
ALDH5A1	Aldehyde dehydrogenase 5 family member A1	9.31	2.97E-87
ACSL5	Acyl-CoA synthetase long chain family member 5	8.64	7.39E-118
ABCC2	ATP binding cassette subfamily C member 2	7.86	8.44E-128
LIPM	Lipase family member M	7.48	2.98E-17
MGAT3	Beta-1,4-mannosyl-glycoprotein 4-beta-N-acetylglucosaminyltransferase	6.90	2.04E-79
ME1	Malic enzyme 1	6.83	5.36E-146
PFKFB1	6-Phosphofructo-2-kinase/fructose-2,6-biphosphatase 1	6.55	2.31E-126
GLYCTK	Glycerate kinase	6.29	2.45E-20
CKMT1A	Creatine kinase, mitochondrial 1A	6.21	3.47E-69
ATP1A4	ATPase Na+/K+ transporting subunit alpha 4	5.95	1.24E-41
GPAM	Glycerol-3-phosphate acyltransferase, mitochondrial	5.20	2.11E-62
PCK2	Phosphoenolpyruvate carboxykinase 2, mitochondrial	5.15	4.56E-159
CD36	CD36 molecule	5.06	5.93E-95
LINCADL	LincRNA adipogenesis and lipogenesis associated	5.02	2.75E-57
CKMT1B	Creatine kinase, mitochondrial 1B	4.87	8.24E-96
ACSS2	Acyl-CoA synthetase short chain family member 2	4.36	3.70E-109
LPL	Lipoprotein lipase	4.26	1.05E-118
PNPLA3	Patatin like phospholipase domain containing 3	4.19	2.28E-93
ACSL4	Acyl-CoA synthetase long chain family member 4	3.77	1.21E-50
SCD	Stearoyl-CoA desaturase	3.71	4.18E-93
FAR2	Fatty acyl-CoA reductase 2	3.69	5.17E-114
SLC2A4	Solute carrier family 2 member 4	3.65	6.23E-76
HMGCS1	3-Hydroxy-3-methylglutaryl-CoA synthase 1	3.50	1.15E-34
CYP4F12	Cytochrome P450 family 4 subfamily F member 12	3.48	1.84E-09
LDHD	Lactate dehydrogenase D	3.46	1.15E-80
ACLY	ATP citrate lyase	3.38	3.14E-87
NMNAT2	Nicotinamide nucleotide adenylyltransferase 2	3.31	1.39E-17
KCNK3	Potassium two pore domain channel subfamily K member 3	3.26	9.34E-95

BBOX1	Gamma-butyrobetaine hydroxylase 1	3.17	9.52E-05
INSIG1	Insulin induced gene 1	3.14	1.13E-63
GSTA1	Glutathione S-transferase alpha 1	3.04	7.16E-26
ECHDC1	Ethylmalonyl-CoA decarboxylase 1	3.02	9.83E-68
SPHK1	Sphingosine kinase 1	2.96	5.45E-53
GK	Glycerol kinase	2.83	2.86E-44
SREBF1	Sterol regulatory element binding transcription factor 1	2.82	1.96E-65
ADCYAP1	Adenylate cyclase activating polypeptide 1	2.81	1.65E-08
LPIN3	Lipin 3	2.79	1.92E-53
ADIPOR2	Adiponectin receptor 2	2.75	3.69E-70
CKMT2	Creatine kinase, mitochondrial 2	2.72	4.96E-48
MGLL	Monoglyceride lipase	2.71	6.71E-64
ELOVL5	ELOVL fatty acid elongase 5	2.58	2.39E-60
ADRB2	Adrenoceptor beta 2	2.58	1.98E-12
NAMPT	Nicotinamide phosphoribosyltransferase	2.55	3.96E-46
FADS2	Fatty acid desaturase 2	2.54	2.25E-53

Supplementary Table S2. List of representative genes downregulated by Capsaicin in ciBAs.

Gene name	Description	Fold Change	Adjusted p-value
ACAN	Aggrecan	-53.52	1.46E-304
ADRA1A	Adrenoceptor alpha 1A	-38.80	2.72E-180
CDH23	Cadherin related 23	-28.29	1.09E-92
PRIMA1	Proline rich membrane anchor 1	-14.72	2.05E-33
ROBO2	Roundabout guidance receptor 2	-14.55	1.56E-30
CNTN1	Contactin 1	-13.50	5.01E-30
GALNT17	Polypeptide N-acetylgalactosaminyltransferase 17	-11.44	1.05E-47
GPRC5B	G protein-coupled receptor class C group 5 member B	-11.03	2.32E-174
PCDH20	Protocadherin 20	-11.02	7.25E-92
TRPV3	Transient receptor potential cation channel subfamily V member 3	-10.66	6.43E-11
NCAM1	Neural cell adhesion molecule 1	-8.77	2.03E-86
TTR	Transthyretin	-7.91	1.51E-68
F2RL2	Coagulation factor II thrombin receptor like 2	-7.20	2.46E-17
CDH20	Cadherin 20	-7.18	1.04E-40
MSR1	Macrophage scavenger receptor 1	-7.06	9.72E-10
FGFR2	Fibroblast growth factor receptor 2	-7.03	5.47E-102
PCDHGA11	Protocadherin gamma subfamily A, 11	-6.82	2.07E-17
TRPV2	Transient receptor potential cation channel subfamily V member 2	-6.69	2.25E-23
TRPM1	Transient receptor potential cation channel subfamily M member 1	-6.61	5.97E-05
AEBP1	AE binding protein 1	-6.59	1.24E-166
P2RY14	Purinergic receptor P2Y14	-6.54	5.64E-14
AQP1	Aquaporin 1 (Colton blood group)	-6.47	1.44E-136
CDH22	Cadherin 22	-5.76	9.49E-09
ST6GAL1	ST6 beta-galactoside alpha-2,6-sialyltransferase 1	-5.65	2.21E-21
BGN	Biglycan	-5.32	2.95E-54
CILP2	Cartilage intermediate layer protein 2	-5.29	2.56E-58
SGCG	Sarcoglycan gamma	-5.21	9.26E-93
MFRP	Membrane frizzled-related protein	-4.79	3.84E-02
MMP11	Matrix metallopeptidase 11	-4.78	3.63E-12
GPC3	Glypican 3	-4.77	4.59E-82
SGCA	Sarcoglycan alpha	-4.70	2.35E-23
POSTN	Periostin	-4.70	9.17E-99
MMP7	Matrix metallopeptidase 7	-4.69	3.62E-44
ADRA2A	Adrenoceptor alpha 2A	-4.48	3.46E-72
MATN2	Matrilin 2	-4.31	5.54E-68
COMP	Cartilage oligomeric matrix protein	-4.29	3.28E-51

ST8SIA4	ST8 alpha-N-acetyl-neuraminide alpha-2,8-sialyltransferase 4	-4.11	2.43E-06
ADAM28	ADAM metallopeptidase domain 28	-4.10	1.98E-06
CSGALNACT1	Chondroitin sulfate N-acetylgalactosaminyltransferase 1	-4.10	1.57E-77
NTN1	Netrin 1	-4.03	1.51E-131
LEPR	Leptin receptor	-3.36	4.22E-68

Supplementary Table S3. Sequences of primers used for qRT-PCR.

Gene	Sense primer	Antisense primer
TBP	ACTACGGGGTTATCACCTGTGAG	GTGCAGGAGTAGGCCACATTAC
UCP1	TCTACGACACGGTCCAGGAG	GAATACTGCCACTCCTCCAGTC
FABP4	GCCAGGAATTTGACGAAGTCA	CCCATTTCTGCACATGTACCAG
MT-CYB	ACTCCACCTCCTATTCTTGCAC	TGTGTAGTAAGGGTGGAAGGTG
MT-ND5	CTTAGGCGCTATCACCACTCTG	CTTGAAGTGGAGAAGGCTACGA
TRPV1	GCTGTCTTCATCATCCTGCTG	TTGCTCTCCTGTGCGATCTTG
TRPV2	TTCCTTTTCGGCTTCGCTGTAG	GCACTGACTCTGTGGCATTG
TRPV3	CTCCTCAACATGCTCATTGCTC	TCTCAAACTCCAAGATGGTCCTG
TRPV4	GCTCGTCTACTTGCTCTTCATG	CAGTTGGTCTGGTCCTCATTG
CIDEA	AAGGCCACCATGTATGAGATGTAC	ACAGGAACCGCAGCAGACTC
LIPG	CACCCTTTATGGCACTAATGCAG	GAAGGTGTTGGTGGCATTCTG
CKMT1	AGCAGGAATGGCTCGAGAC	ATCCTCCTCATTCACCCAGATC
CD36	CAACCTATTGGTCAAGCCATCAG	TGTTTGCCTTCTCATCACCAATG
SCD	ACTTGTCTGACCTAGAAGCTGAG	GATGAAGCACATCATCAGCAAG
KCNK3	CCATCACCGTCATCACCAC	CAGCGCGTAGAACATGCAG
SREBF1	CCTGGTCTACCATAAGCTGCAC	CTGCACACTCTGCCAGGTTC
FASN	CCTTCGAGGTGTCAGAGAAC	TTTCCGGGTGGTCGAAGAG
PLIN1	AGCATTGAGAAGGTGGTGGAG	ACTTCTGGGCTTGCTGGTG
LIPE	CTCAGTGTGCTCTCCAAGTG	CTTTCTGGTCTGAGTTGGAGTG
ADIPOQ	CTGGTGAGAAGGGTGAGAAAG	GTTTCACCGATGTCTCCCTTAG
AEBP1	AACGCCACCATCTCTGTGAG	CAAGATTCGCCAGTAATCACCAC
CEBPA	AACATCGCGGTGCGCAAGA	AGCTCCAGCACCTTCTGCT
PPARGC1A	AGGTCAAGATCAAGGTCTCCAG	GGTGTCTGTAGTGGCTTGACTC
PPARG	TGGAATTAGATGACAGCGACTTG	CTTCAATGGGCTTCACATTCAG
RXRG	GGCCTACACCAAGCAGAAGTATC	CAAGCCAATGGAACGCAGAG
GK	GATTTGTCTGCCGTCACGATG	CCAACCCATTGACTTCATCACAG
GPAM	GATCGCATTATCGAAGGTCACTAC	TTGCTACACTCCACAGGCTCTC
AGPAT2	CCCGTGGTGTACTCTTCCTTC	TTCCAGCACCTGCACTGTGA
LPIN3	CATCCTTCTGTCTCCCAGCAG	GGCGACCTTGAACACCTCTG
DGPAT2	TACTCCAAGCCCATCACCAC	ATGGTGTGGTACAGGTCGATG
AQP7	CTGCCACCATCTACAGTCTCTTC	TCATGTGATCAGGAAGGTAGGTG
GPD1	CTGACCTGATCACTACCTGCTATG	GCAACTCTTTCTCCAGCTGCTC
LXRA	CAGATTGCCCTGCTGAAGAC	AGGGTTGTACCTCCGAGATG
RORC	GAGGATGAGATTGCCCTCTACAC	GGCCAGCTCCAGATTGTACTG
NR4A2	AACTTGCAGAATATGAACATCGAC	GTTCTTCCACTCTCTTGGGTTC
NR4A3	TTGGAGCTGTTTGTCCTCAGAC	ACTGAAGTCGATGCAGGACAAG
